# Down regulation of lactate dehydrogenase initiates apoptosis in HeLa and MCF-7 cancer cells through increased voltage-dependent anion channel protein and inhibition of BCL2

**DOI:** 10.18632/oncotarget.27950

**Published:** 2021-04-27

**Authors:** Suhail Al-Salam, Karthishwaran Kandhan, Manjusha Sudhadevi

**Affiliations:** ^1^Department of Pathology, College of Medicine & Health Sciences, United Arab Emirates University, AlAin, UAE

**Keywords:** cancer metabolism, LDHA, apoptosis, VDAC

## Abstract

Malignant cells commonly use aerobic glycolysis for ATP production; this is known as the Warburg effect, where pyruvate is converted to lactate, by enzyme lactate dehydrogenase A (LDH-A). In this study, we have investigated the effect of inhibition of LDH-A on cells viability and identifying the mechanism of cell death in HeLa and MCF-7 cancer cells.

Human cervical cancer HeLa cell line and breast cancer MCF-7 cell line were used to investigate the effect of inhibition of LDH-A by sodium oxamate on cell survival and proliferation using western blot, spectrophotometry, and immunofluorescent study.

There was significant reduction in LDH-A (*P* < 0.001) and cell viability (*P* < 0.001) in a dose-dependent mode in both HeLa and MCF-7 SO-treated cancer cells. The voltage-dependent anion channel (VDAC) protein was significantly increased (*P* < 0.001) in association with decreased LDH-A. The proapoptotic proteins; cytochrome C (*P* < 0.001), BAX (*P* < 0.001), cleaved caspase-3 (*P* < 0.001), cleaved caspase-8 (*P* < 0.001), and cleaved caspase-9 (*P* < 0.001) were significantly increased in association with decreased LDH-A. While, the anti-apoptotic protein Bcl2 was significantly decreased (*P* < 0.001) in association with decreased LDH-A.

We conclude that Inhibition of LDH-A can decrease cells viability through activation of intrinsic apoptotic pathway via increased VDAC protein and inhibition of Bcl2 as well as activation of the extrinsic apoptotic pathway through activation of caspase-8.

## INTRODUCTION

Malignant cells commonly use aerobic glycolysis for ATP production; this is known as the Warburg effect [1]. Pyruvate is converted to lactate by enzyme lactate dehydrogenase A (LDH-A) where nicotinamide adenine dinucleotide (NAD+) is generated from NADH in this process [2]. The production of NAD^+^ is required by glyceraldehyde 3-phosphate dehydrogenase to maintain glycolysis and ATP production [2]. LDH, which owns two subunits LDH-A and LDH-B, is an enzyme commonly existing in human cells [3]. LDH-A catalyzes the conversion of pyruvate to lactate with the liberation of NAD^+^, which possesses a vital role in glycolysis. It has long been noted that LDH- A expression is upregulated in human neoplastic tissues [3].

Recently, many studies have shown that LDH-A plays a vital role in maintaining tumor growth and progression [4]. Furthermore, relevant studies have demonstrated that inhibition of LDH-A induces oxidative stress and suppresses tumor growth in a variety of cancer cell lines [5, 6].

Since aerobic glycolysis is the favored way of ATP production in cancer cells, it becomes a very attractive target for cancer therapies [7].

Sodium oxamate (SO) is a competitive inhibitor of LDH-A, hence, we use SO to block aerobic glycolysis and reduces the main energy source in cancer cells [8]. However, the detailed mechanism remains largely unclear. In this study we will investigate changes in apoptotic and oxidative stress pathways in association with the use of sodium oxamate in cervical (HeLa) and breast (MCF-7) cancer cell lines aiming in identifying mechanism of cancer cell death following SO treatment.

## RESULTS

### Effect of sodium oxamate on cell proliferation

**Figure 1:**
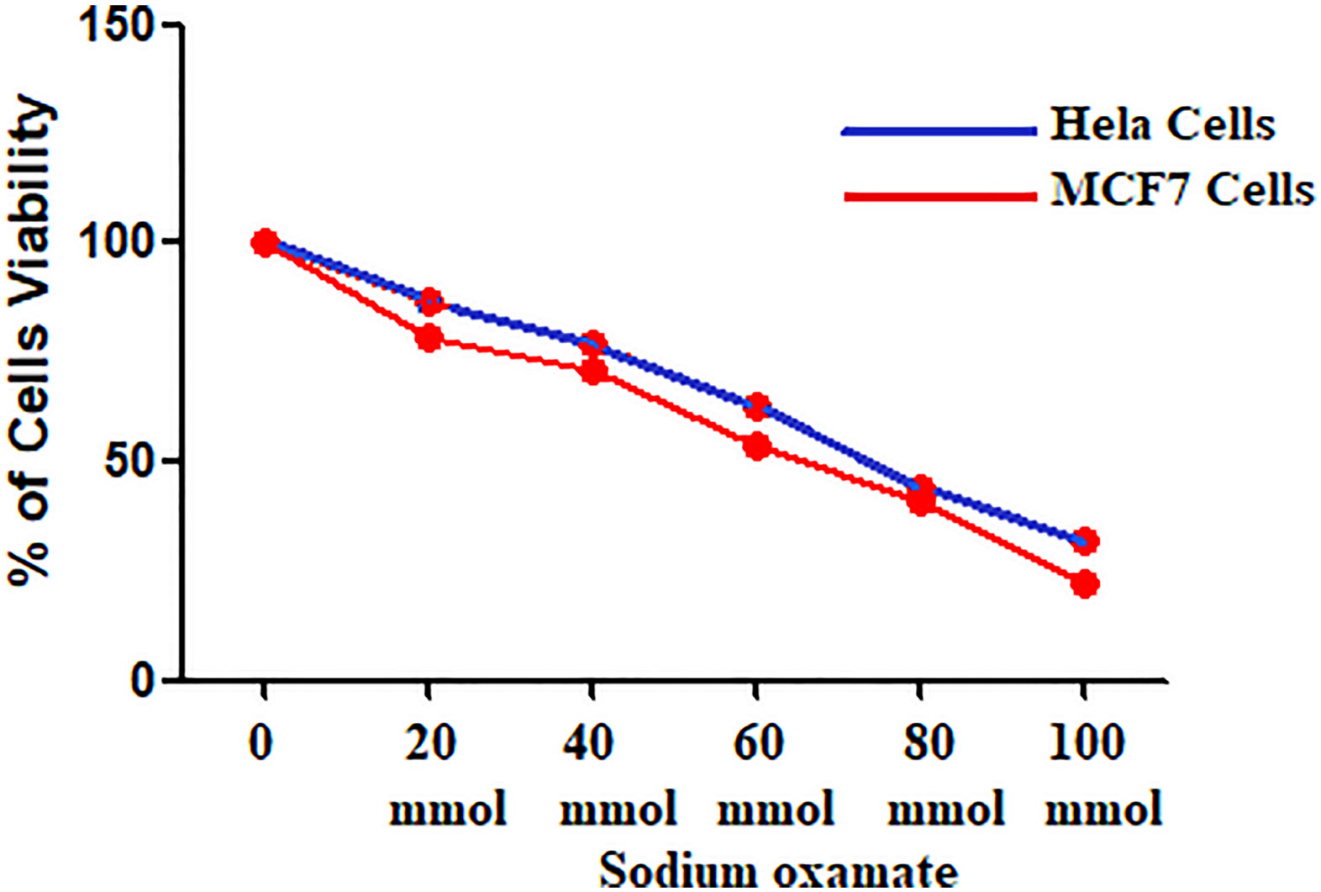
HeLa and MCF-7 Cell viability at different concentrations of sodium oxamate.

**Figure 2:**
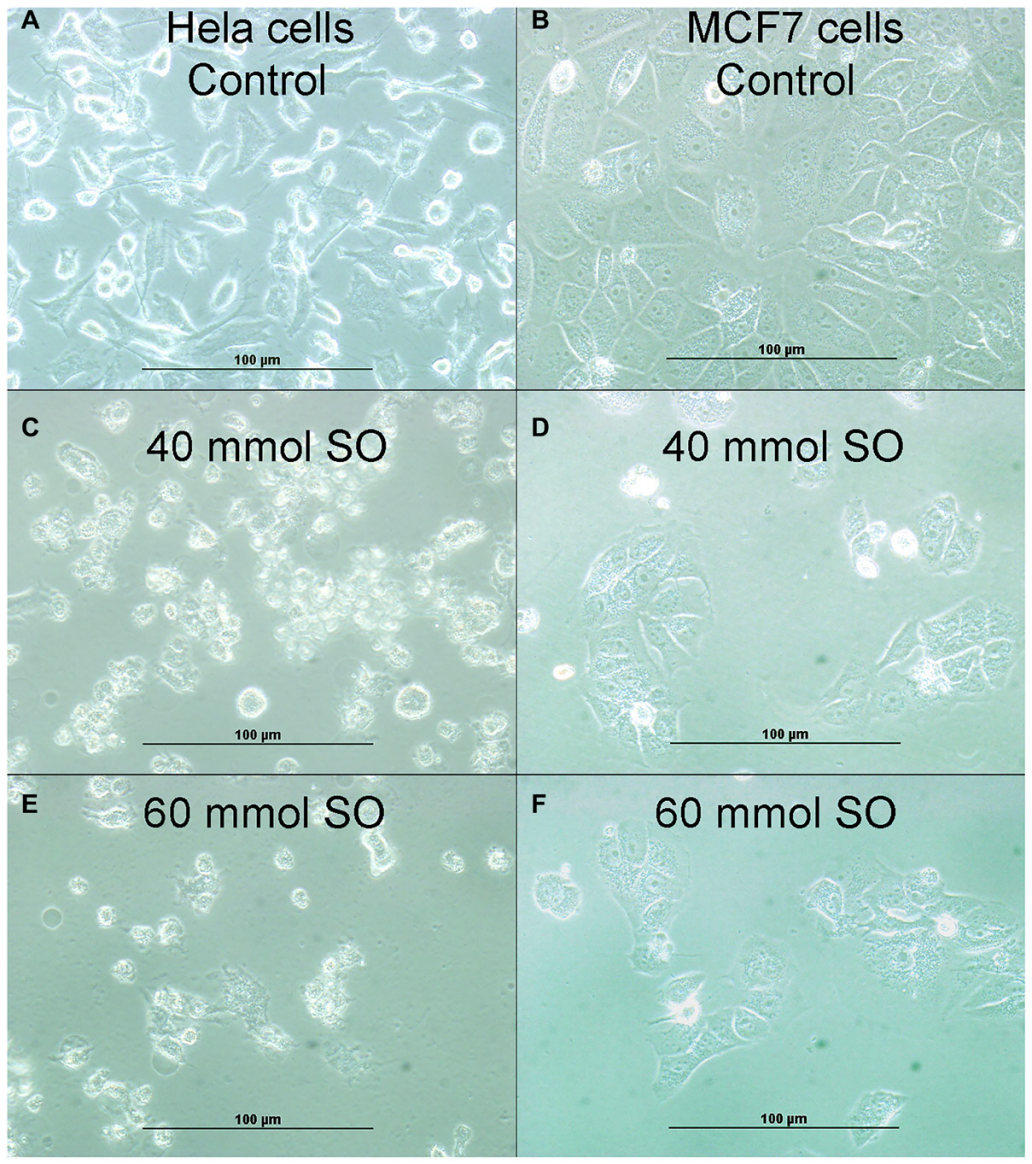
(**A**) Showing cellular growth of untreated HeLa cells. (**B**) Showing cellular growth of untreated MCF-7 cells. (**C**) Showing reduction of cellular proliferation in 40 mmol-treated HeLa cells. (**D**) Showing reduction of cellular proliferation in 40 mmol-treated MCF-7 cells. (**E**) Showing more reduction of cellular proliferation in 60 mmol-treated HeLa cells. (**F**). Showing more reduction of cellular proliferation in 60 mmol-treated MCF-7 cells.

The effect of SO on cell proliferation was determined by MTT assay (Figure 1). The proliferation of HeLa and MCF-7 cells was significantly inhibited by various concentration of SO (20–100 mmol/L). The inhibitory effect was observed after 24-hour of incubation. Figure 1 reveals treatment with SO can lead to significant inhibition of proliferation. The SO inhibitory effect was dose-related. The treatment of 60 mmol/L of SO revealed almost 40% cell inhibition on both HeLa and MCF-7 cells (Table 1). Hence, we have selected the 40 and 60 mmol/L concentrations of SO for further analysis. Treatment with SO has shown dose-related inhibition of cell growth of both HeLa cells and MCF-7 cells (Figures 1 and 2).

**Table 1:** HeLa and MCF-7 cells viability at different concentrations of sodium oxamate

Concentration (mmol/L)	HeLa Cell Viability	*P* Value	MCF-7 Cell Viability	*P* Value
0	100		100	
20	78.3 ± 1.35	< 0.001	86.4 ± 1.8	< 0.001
40	71.3 ± 0.9	< 0.001	76.7 ± 0.9	< 0.001
60	53.9 ± 0.81	< 0.0001	62.9 ± 0.9	< 0.0001
80	40.8 ± 0.72	< 0.0001	44.29 ± 0.6	< 0.0001
100	22.6 ± 0.4	< 0.0001	32.06 ± 0.36	< 0.0001

### Changes in the activities of antioxidant enzymes

#### SO reduces superoxide dismutase (SOD) activity

**Figure 3:**
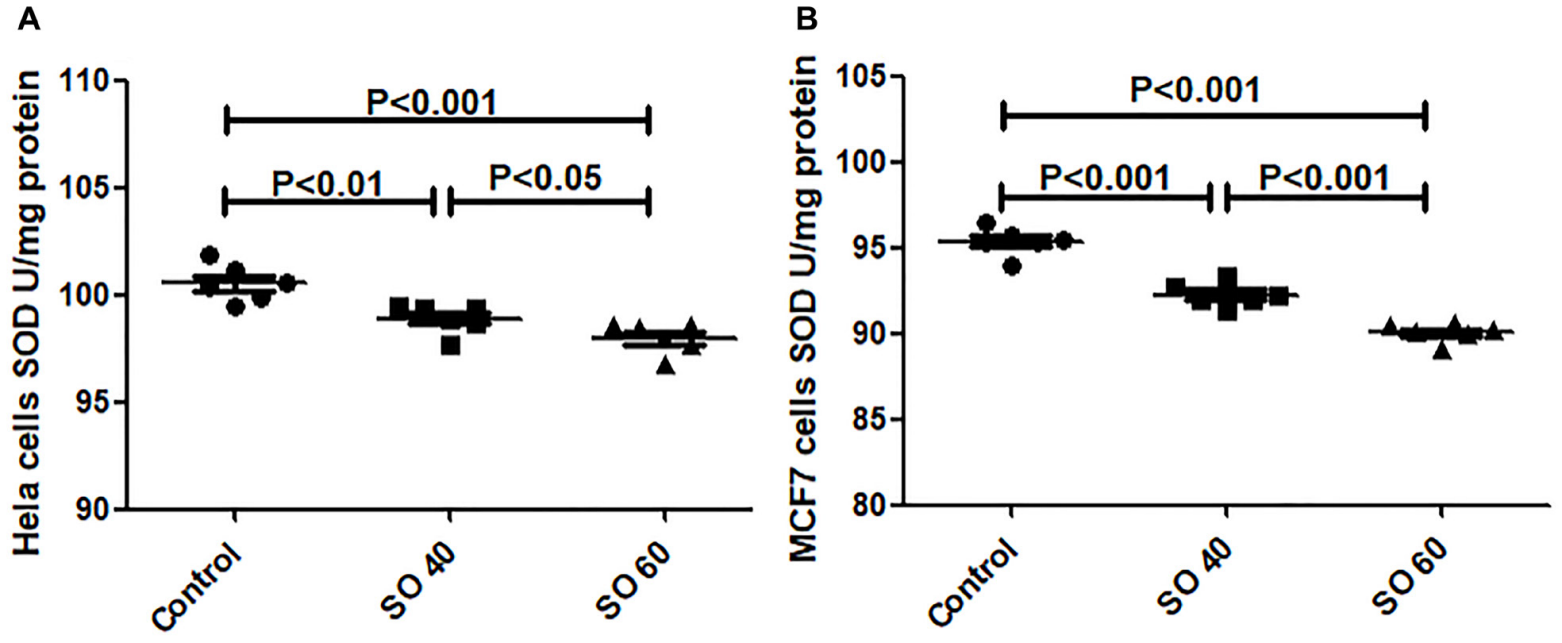
Anti-oxidant activity of SOD in SO-treated HeLa (**A**) and MCF-7 (**B**) cell lines.

SO (40 and 60 mmol/L) treatment significantly reduced the activity of SOD in HeLa and MCF-7 cells, when compared with untreated control cells (Figure 3). The two tested doses, 40 and 60 mmol/L of SO, significantly decreased SOD levels in both HeLa and MCF7 cancer cells, suggesting that SO increased reactive oxygen species (ROS) content in both HeLa and MCF7 cancer cells.

#### SO decreases reduced glutathione (GSH)

**Figure 4:**
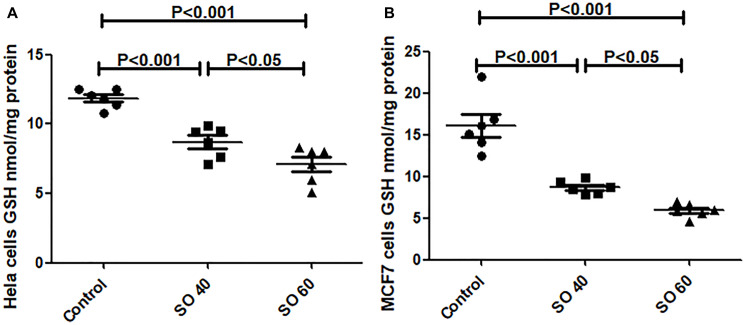
GSH levels in SO- treated HeLa (**A**) and MCF-7 (**B**) cancer cell lines.

Reduced GSH is an important cellular antioxidant through donating thiol groups. There was a significant reduction of reduced GSH levels in SO treated HeLa and MCF7 cancer cells (Figure 4). The two tested doses, 40 and 60 mmol/L of SO, significantly decreased GSH levels in both HeLa and MCF7 cancer cells, suggesting that SO increased ROS content in both HeLa and MCF7 cancer cells.

#### Changes in the LDH-A level

**Figure 5:**
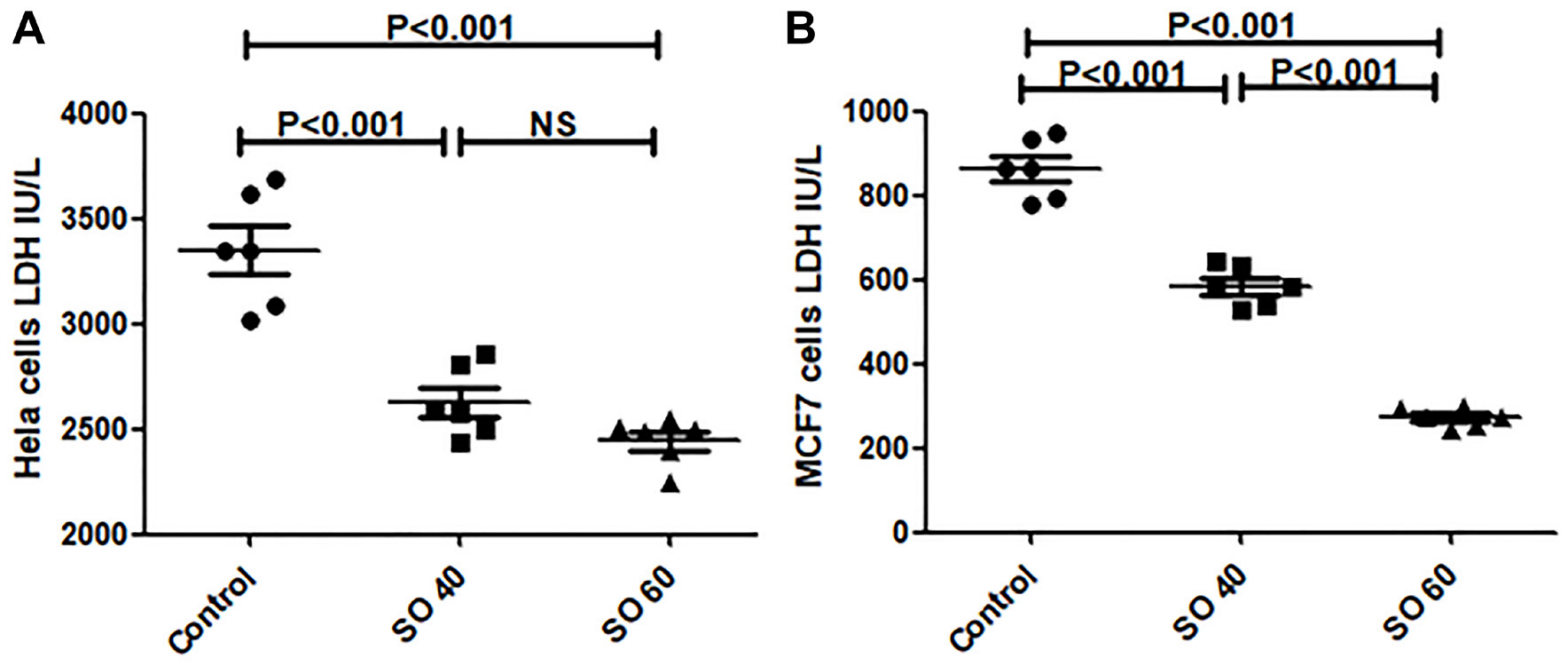
Sodium Oxamate inhibits LDH enzyme activity in HeLa (**A**) and MCF-7 (**B**) cancer cells. Cells were treated with 0, 40, 60 mM sodium oxamate for 24 hour.

In this study, LDH-A was significantly decreased in SO- treated groups when compared to control cancer cells in both HeLa and MCF-7 (Figure 5). We have noticed LDH-A reduction is even greater in 60 mmol/L compared to 40 mmol/L treated groups, suggesting a dose-response activity of SO on LDH-A. Accordingly, SO treatment significantly inhibited LDH-A activity in cancer cells, while ROS content was distinctly increased.

### Correlation between the expression of LDH-A and VDAC

**Figure 6:**
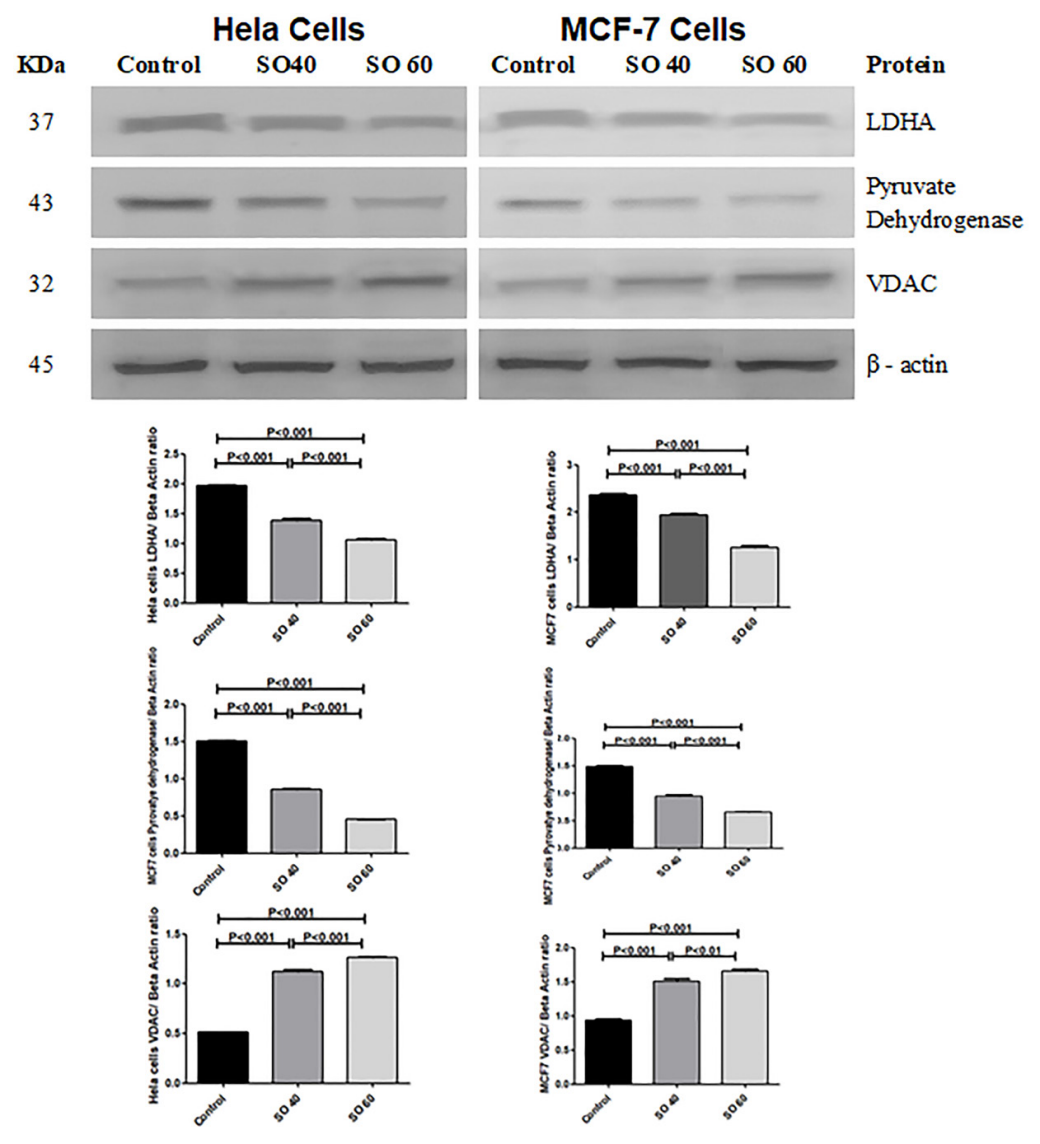
Expression of LDHA, PDH and VDAC proteins in 40 mmol and 60 mmol SO treated HeLa and MCF-7 cancer cells compared to control non-treated HeLa and MCF-7 cancer cells.

The effect of SO on the expression pattern of VDAC, LDH-A and pyruvate dehydrogenase (PDH) in HeLa and MCF-7 cells was analyzed by western blot. The expressions of VDAC was found to be significantly increased in 40 and 60 mmol SO- treated HeLa and MCF-7 cells when compared to control cells. Whereas, similar concentrations of SO had shown significant decrease in the expression of LDH-A and PDH when compared to control non-treated HeLa and MCF-7 cancer cells (Figure 6).

### Expression of apoptotic and anti-apoptotic proteins in relation to decreased LDH-A

**Figure 7:**
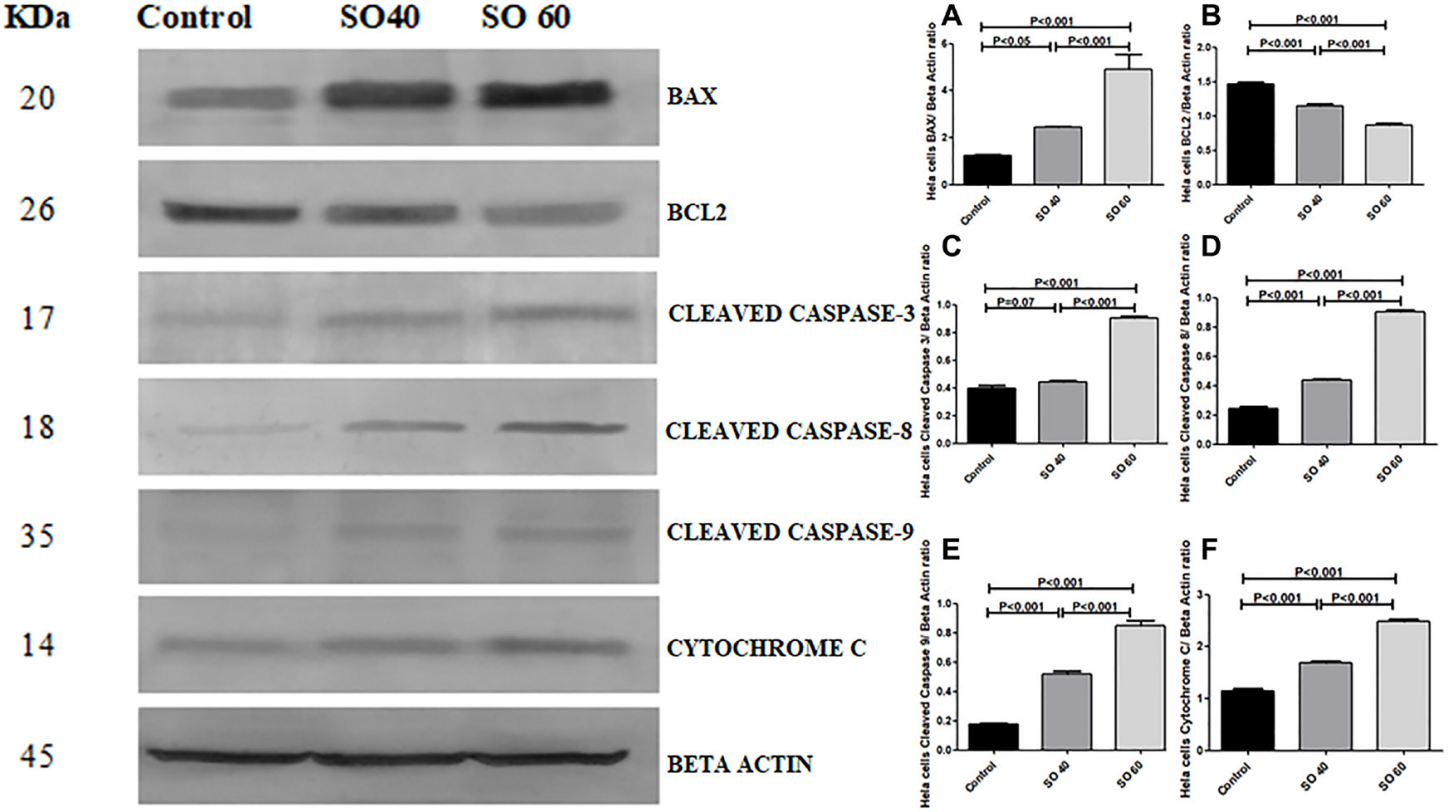
Expression of apoptotic proteins Bax, cleaved caspase-3, cleaved caspase-8, cleaved caspase-9, cytochrome c and anti-apoptotic proteins Bcl2 in 40 mmol and 60 mmol SO treated HeLa cancer cells compared to control non-treated HeLa cells.

**Figure 8:**
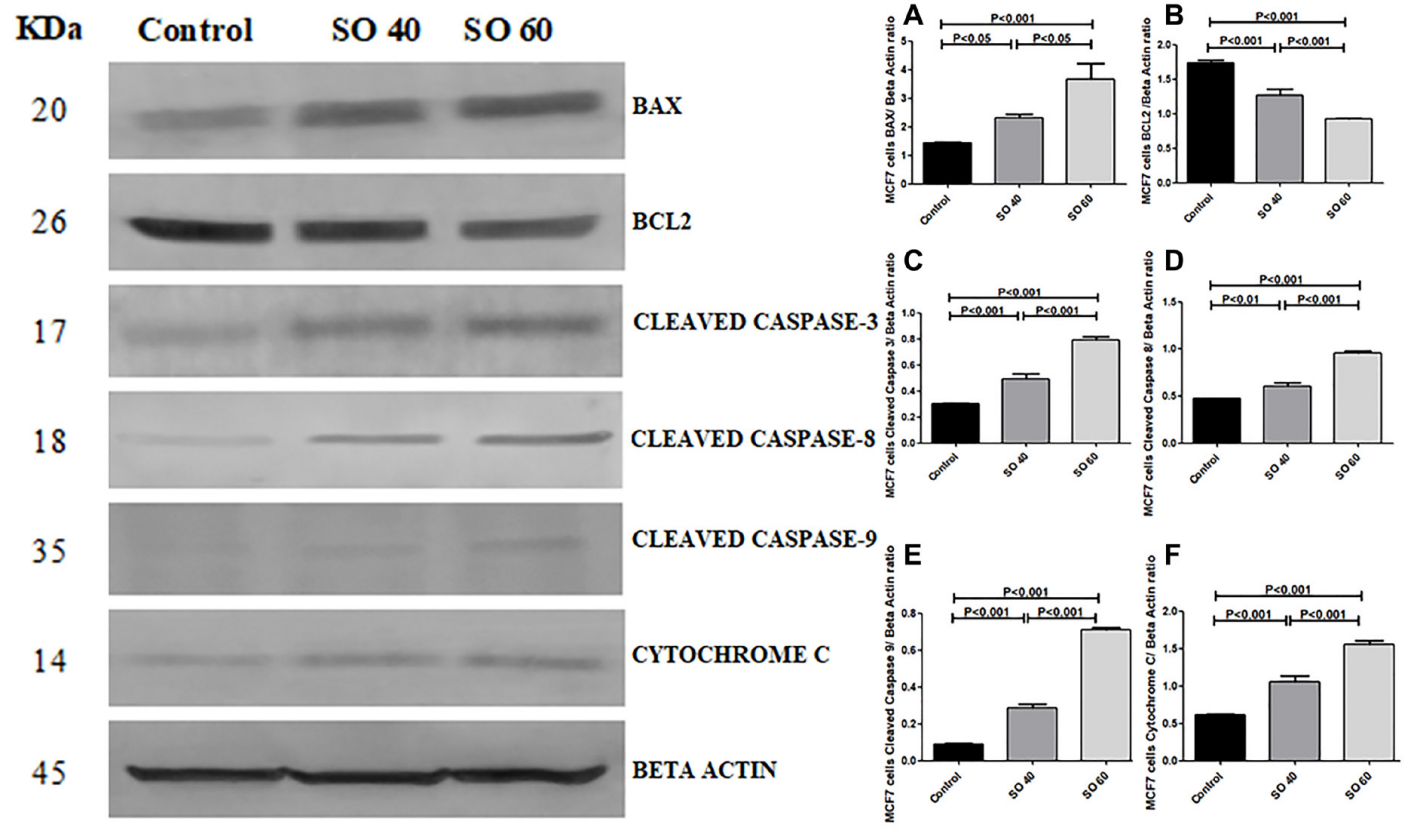
Expression of apoptotic proteins Bax, cleaved caspase-3, cleaved caspase-8, cleaved caspase-9, cytochrome c and anti-apoptotic proteins Bcl2 in 40 mmol and 60 mmol SO treated MCF-7 cancer cells compared to control non-treated MCF-7 cells.

**Figure 9:**
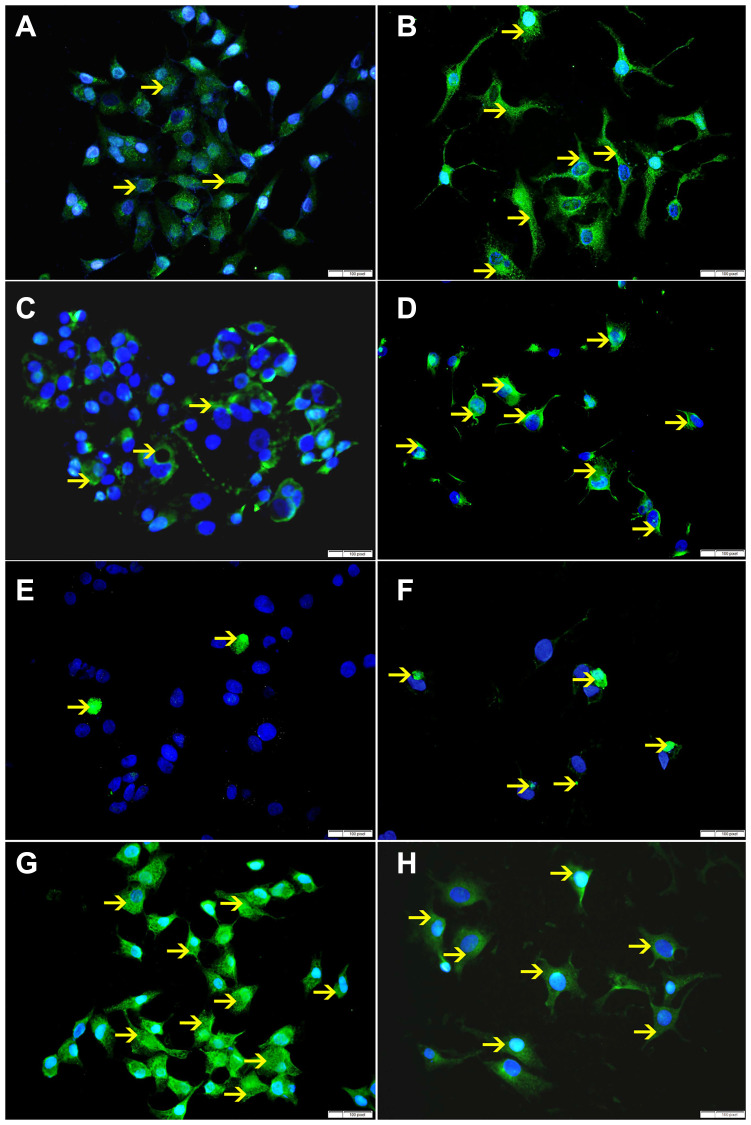
(**A**) Showing low cytoplasmic expression of Bax by non-treated HeLa cells (arrow). (**B**) Showing higher cytoplasmic expression of Bax by SO 60mmol treated HeLa cells (arrow). (**C**) Showing low cytoplasmic expression of VDAC by non-treated HeLa cells (arrow). (**D**) Showing higher cytoplasmic expression of VDAC by SO 60 mmol treated HeLa cells (arrow). (**E**) Showing few apoptotic bodies expressing cleaved caspase-3 in non-treated HeLa cells (arrow). (**F**) Showing higher number of apoptotic bodies expressing cleaved caspase-3 in SO 60mmol treated HeLa cells (arrow). (**G**) Showing high cytoplasmic expression of Bcl-xL by non-treated HeLa cells (arrow). (**H**) Showing lower cytoplasmic expression of Bcl-xL by SO 60 mmol treated HeLa cells (arrow).

**Figure 10:**
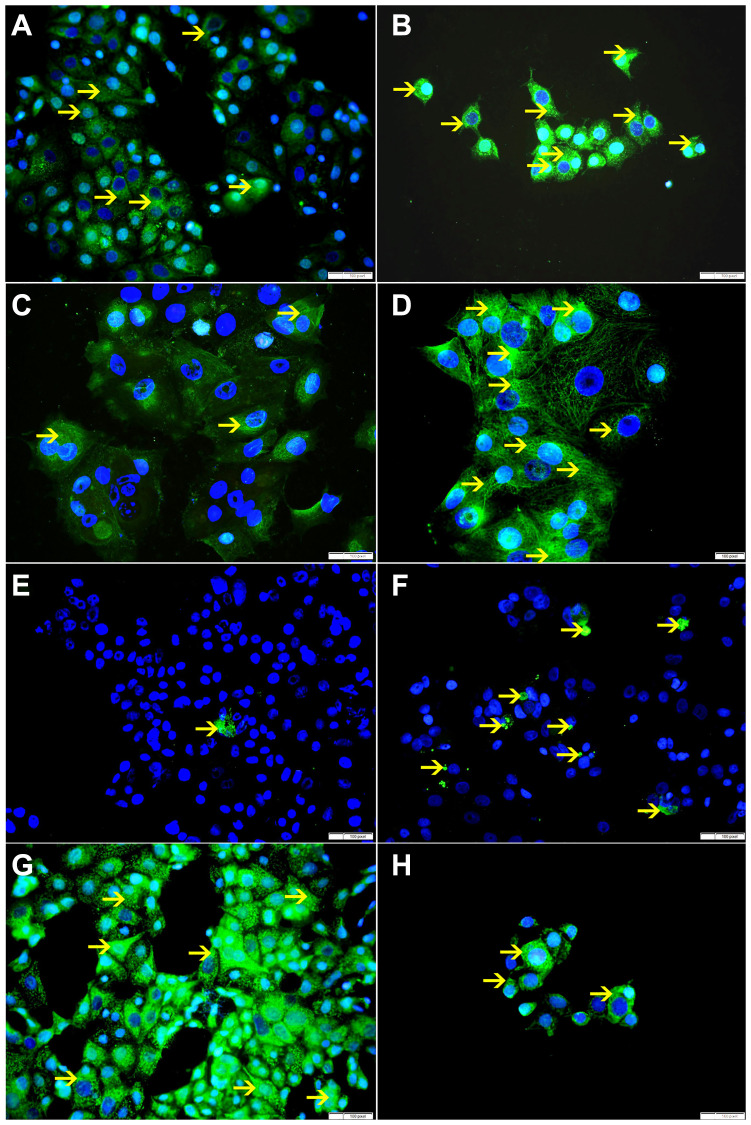
(**A**) Showing cytoplasmic expression of Bax by non-treated MCF-7 cells (arrow). (**B**) Showing higher cytoplasmic expression of Bax by SO 60 mmol treated MCF-7 cells (arrow). (**C**) Showing low cytoplasmic expression of VDAC by non-treated MCF-7 cells (arrow). (**D**) Showing higher cytoplasmic expression of VDAC by SO 60 mmol treated MCF-7 cells (arrow). (**E**) Showing few apoptotic bodies expressing cleaved caspase-3 in non-treated MCF-7 cells (arrow). (**F**) Showing higher number of apoptotic bodies expressing cleaved caspase-3 in SO 60mmol treated MCF-7 cells (arrow). (**G**) Showing high cytoplasmic expression of Bcl-xL by non-treated MCF-7 cells (arrow). (**H**) Showing lower cytoplasmic expression of Bcl-xL by SO 60 mmol treated MCF-7 cells (arrow).

The effect of SO on the expression pattern of cleaved caspase 3, cleaved caspase 8, cleaved caspase 9, Bax, cytochrome c, and Bcl-2 in HeLa and MCF-7 cells was analyzed by western blot. The expressions of cleaved caspase 3, cleaved caspase 8, cleaved caspase 3, Bax, and cytochrome C, proteins were found to be significantly increased in 40 mmol and 60 mmol SO treated HeLa and MCF-2 cells when compared to control non-treated HeLa and MCF-7 cells. Whereas, similar concentrations of SO had shown significant decrease in the expression of Bcl-2 in treated HeLa and MCF-7 cells when compared to control non-treated HeLa and MCF-7 cells (Figures 7–10).

### Expression of proliferative markers in relation to SO treatment

**Figure 11:**
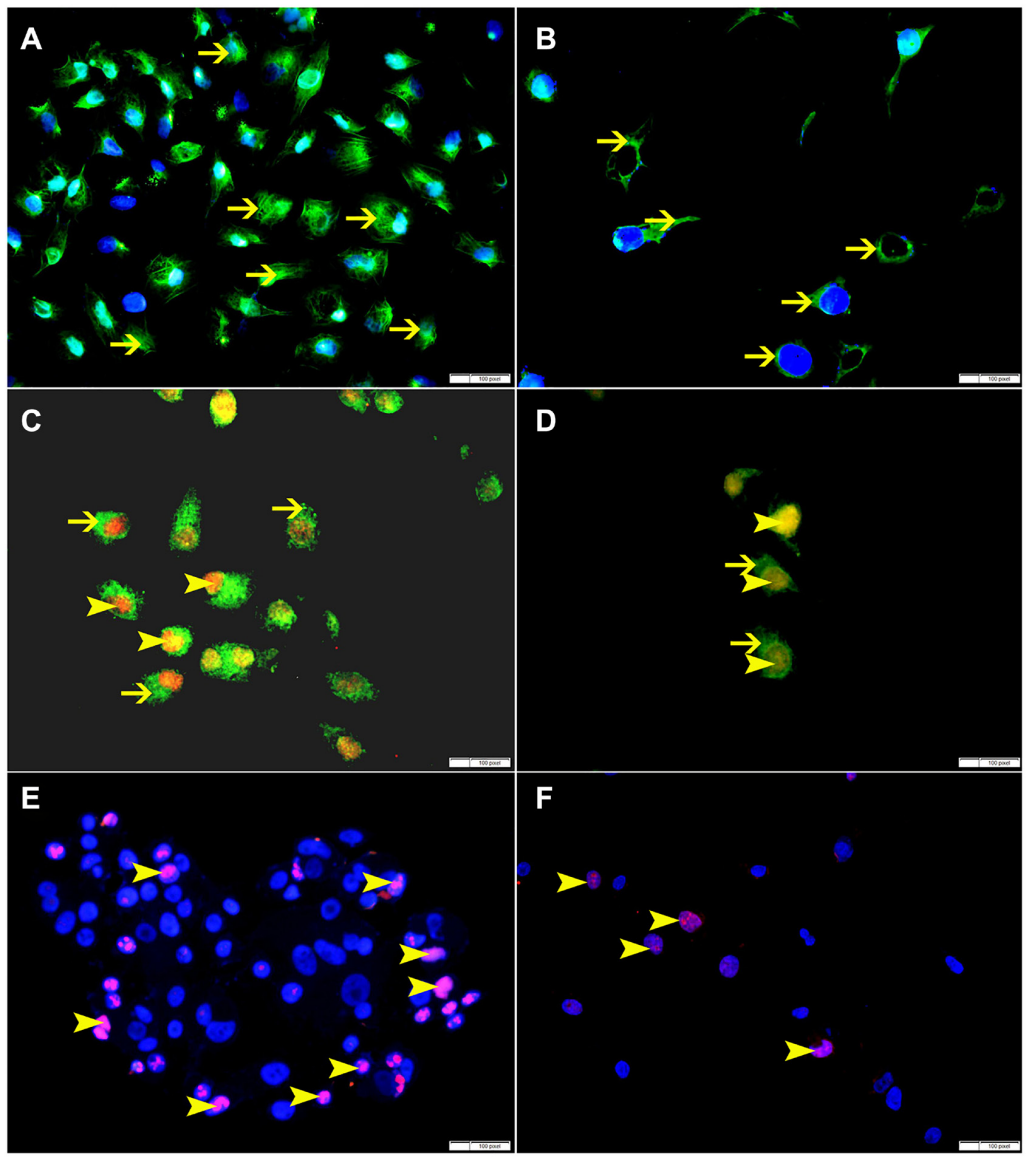
(**A**) Showing higher cytoplasmic expression of p-NFKB by non-treated HeLa cells (arrow). (**B**) Showing lower cytoplasmic expression of p-NFKB by SO 60 mmol treated HeLa cells (arrow). (**C**) Showing higher cytoplasmic expression of VEGF (thin arrow) and nuclear expression of BMI-1 (arrowhead) by non-treated HeLa cells. (**D**) Showing lower cytoplasmic expression of VEGF (thin arrow) and nuclear expression of BMI-1 (arrowhead) by SO 60 mmol treated HeLa cells. (**E**) Showing higher number of cells expressing ki-67 in non-treated HeLa cells (arrow). (**F**) Showing lower number of cells expressing ki-67 in SO 60 mmol treated HeLa cells (arrow).

**Figure 12:**
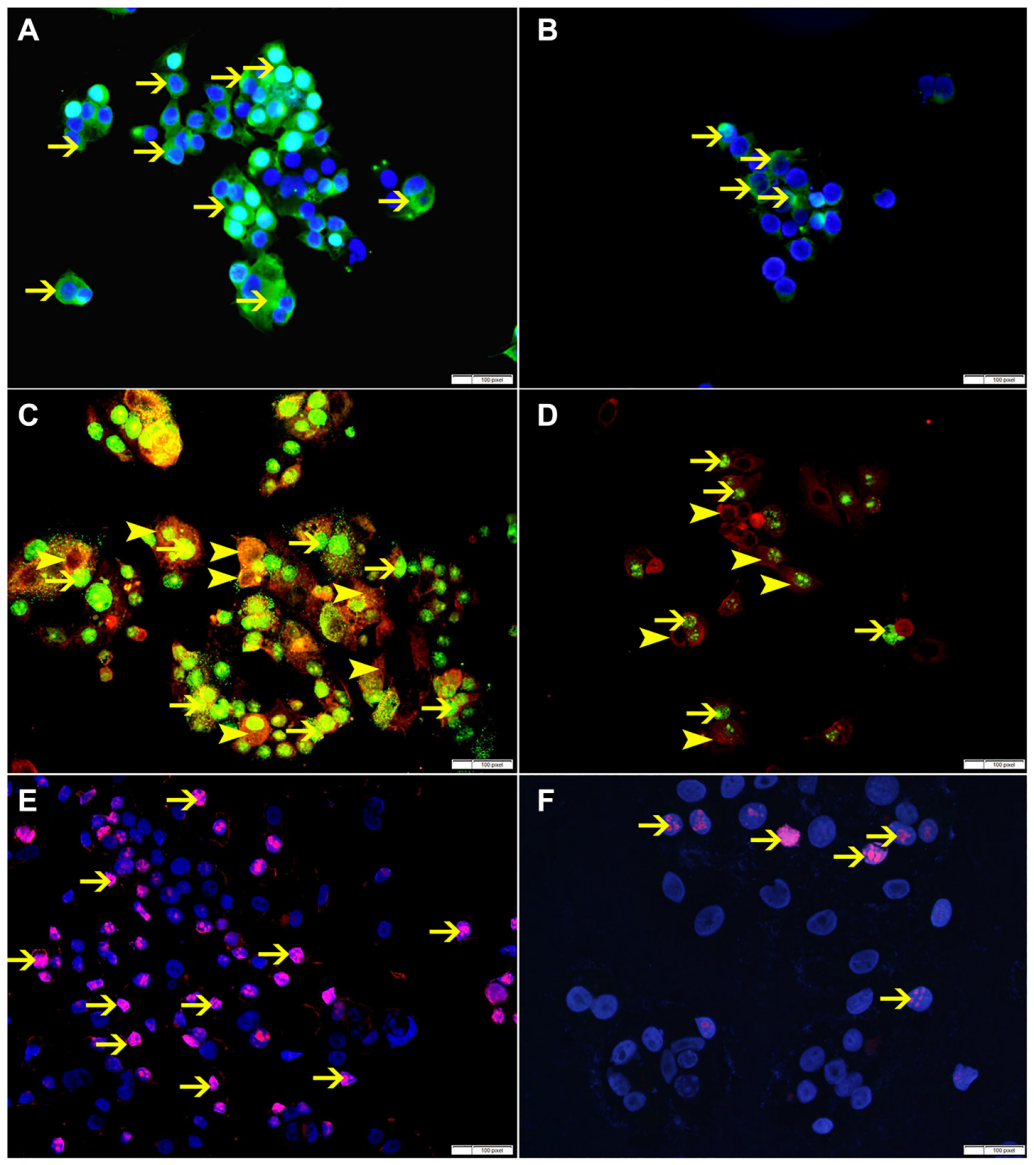
(**A**) Showing high cytoplasmic expression of p-NFKB by non-treated MCF-7 cells (arrow). (**B**) Showing lower cytoplasmic expression of p-NFKB by SO 60 mmol treated MCF-7 cells (arrow). (**C**) Showing higher cytoplasmic expression of VEGF (arrowhead) and nuclear expression of BMI-1 (thin arrow) by non-treated MCF-7 cells. (**D**) Showing lower cytoplasmic expression of VEGF (arrowhead) and nuclear expression of BMI-1 (thin arrow) by SO 60 mmol treated MCF-7 cells. (**E**) Showing higher number of cells expressing ki-67 in non-treated MCF-7 cells (arrow). (**F**) Showing lower number of cells expressing ki-67 in SO 60 mmol treated MCF-7 cells (arrow).

SO treatment has shown decrease in the expression of p-NFK-B, VEGF, BMI-1 and ki-67 at 60 mmol concentration, suggesting that SO can reduce cellular proliferation (Figures 11, 12).

## DISCUSSION

Cancer cells produce a large amount of energy through glycolysis even in the presence of sufficient oxygen [1–3]. LDH-A, a key regulator of glycolysis, reversibly catalyzes the conversion of pyruvate to lactate. In this study, we show SO significantly reduces LDH-A in a dose-dependent mode.

We also show SO significantly inhibits the viability of human cervical (HeLa) and breast (MCF-7) cancer cells in a dose-dependent fashion.

Increased levels of LDH-A have been reported in several types of cancers [9–11], and their levels of expression have been correlated with clinical stages in esophageal, pancreatic, prostate carcinomas; suggesting that overexpression of LDH-A has proliferative role on cancer cells. These studies [9–11] further support the concept that therapies targeting LDH-A may provide useful strategies in controlling cancer progression. Our immunofluorescent staining results have also shown a decrease in the expression of growth signals markers; p-NFK-B, BMI-1, VEGF and ki-67 in SO treated HeLa cells and MCF-7 cells suggesting an association with the decreased LDH-A in these cells.

In addition, we have shown treatment with SO significantly augments ROS production leading to a significant reduction in SOD and GSH levels in SO-treated cancer cells suggesting that inhibition of LDH-A plays an important role in the induction of ROS. Zhao Z et al. have also shown that SO treatment significantly augments ROS production [12]. Reports have shown increased ROS can induce apoptosis [13–15].

We have also shown SO significantly increases proapoptotic proteins BAX and cytochrome c. In addition, there is significant increase in the initiating active caspases; cleaved caspase 8 and 9 and terminal executer cleaved caspase 3 which is accompanied by significant reduction in antiapoptotic protein Bcl2. These findings support apoptotic role of SO. Chandan et al. have shown inhibition of LDH-A can lead to apoptosis, which supports our finding [16].

Interestingly, we also show SO-treated cancer cells have significant increase in VDAC protein. VDAC protein has also been shown to play an important role in apoptosis [17]. Following the initiation of apoptotic signals there is increased permeability of VDAC, which allows the release of proapoptotic proteins such as cytochrome c from the mitochondria. Although cytochrome c plays a crucial role in oxidative phosphorylation within the mitochondria, in the cytosol it activates caspases, which play a major role in programed cell death [18]. While the mechanism of VDAC-enabled cytochrome c release has not yet been fully understood, there have been reports suggest that oligomerization between individual subunits can create a large stretchy pores through which cytochrome c can pass [19]. Remarkably, the release of cytochrome c is also regulated by Bax, which interacts directly with VDAC to increase pore size and promote cytochrome c release, whereas anti-apoptotic Bcl2 produces the opposite effect [20]. In fact, it has been shown that antibodies which are capable of inhibiting VDAC also interfere with Bax-mediated cytochrome c release [21].

Zalk et al. have suggested that VDAC overexpression can lead to increased cell death since higher VDAC expression favors VDAC oligomerization [19]. Moreover, it has been advocated that oligomeric VDAC mediates the release of cytochrome c [19], because the internal diameter of a single VDAC pore is 2.5-3.0 nm, which is insufficient to pass a folded protein [22]. The increased VDAC has been shown to assemble to form polymers that increases the diameter of VDAC and allows the release of cytochrome c [22]. Reports have shown upregulation of Bcl2 prevents VDAC oligomerization and then apoptosis [23, 24].

Our study has shown for the first time a significant dose-related increase in VDAC protein in SO-treated HeLa and MCF7 cells as compared with control cancer cells; suggesting SO has a crucial effect on VDAC production either directly or through the increase in free radicals or a decrease in LDH-A.

The increase in VDAC can lead to increase in cell death through activation of the intrinsic pathway of apoptosis by enhancing the release of cytochrome c and inactivation of Bcl2, which may be the main mechanism of apoptosis in our study [19].

We have also shown a significant increase in cleaved caspase-8 suggesting activation of extrinsic pathway of apoptosis in SO-treated HeLa and MCF7 cancer cells. There was no previous reports on activation of extrinsic pathway of apoptosis in association with SO-treated cancer cells. It is possible that the activation of caspase-8 is related to increase in free radical production associated with SO treatment. This is supported by other studies, which also show activation of caspase-8 by oxidative stress [25, 26].

In conclusion, the Inhibition of LDH-A can decrease cells viability through activation of intrinsic apoptotic pathway via increased VDAC protein and inhibition of Bcl2 as well as activation of the extrinsic apoptotic pathway through activation of caspase-8.

## MATERIALS AND METHODS

### Chemicals

Sodium oxamate was supplied by Sigma-Aldrich (St. Louis, MO, USA). All other chemicals were procured from Merck (analytical grade).

### Cell culture

The human cervical cancer cell line (HeLa) and breast cancer cell line (MCF-7) were obtained from ATCC, Rockville, MD, USA. Entire cell culture experiments were conducted using pre-sterile consumables and followed aseptic techniques already in place. The HeLa cell was grown as a monolayer in Dulbecco’s Modified Eagle’s Medium (DMEM) and the MCF-7 cell was grown as a monolayer in MEM with 10% FBS, 200 mM L-glutamine, 10,000 U/mL penicillin, and 10 mg/mL streptomycin at 37°C in 5% CO_2_. Cells were harvested by trypsinization once 80% confluency was achieved during subculture. Stocks were maintained in 25 cm^2^ tissue culture flasks.

### MTT (3-(4,5-dimethylthiazol-2-yl)-2,5-diphenyltetrazolium bromide) assay

The MTT assay, which detects mitochondrial dehydrogenase activity in living cells, was used to assess cytotoxicity [27]. Briefly, cells (5 × 10^3^ cells/well) plated in 96-well flat-bottom plates were exposed with an increasing range of concentration of SO. After incubation, 10 μl of MTT (5 mg/ml) in PBS were added to each well and allowed to develop color. DMSO was added to stop the reaction and the absorbance at 570 nm was read using Tecan infinite microplate reader.

### Cell treatments

Both HeLa and MCF-7 cells were treated with SO in different concentrations and incubated at 37°C in 5% CO_2_ incubator. After 24 h incubation, the cells were harvested by trypsinization for further experiments.

Group I: Control (Untreated HeLa/MCF-7 cells)

Group II: HeLa/MCF-7 cells + SO (40 mmol/L)

Group III: HeLa/MCF-7 cells + SO (60 mmol/L)

### Biochemical estimations

The cells were harvested by trypsinization. The obtained cell pellet was suspended in PBS and that suspension was used for biochemical estimations. Superoxide dismutase (SOD) was assayed by the method of Kakkar et al. [27]. Nicotinamide adenine dinucleotide (NADH), phenazine methosulphate, and nitroblue tetrazolium formazon formation are all inhibited in this assay. The reaction was initiated by the addition of NADH. For a set period of time, a known amount of enzyme preparation was permitted to react with H_2_O_2_ in the presence of glutathione (GSH). The GSH material that remained after the reaction was then calculated. The total GSH content was measured based on the development of a yellow color when 5,5′-dithiobis-2-nitrobenzoic acid was added to compound containing sulfhydryl groups [28].

### Lactate dehydrogenase A activity

LDH-A Assay kit (Sigma, USA) was used to determine the intracellular LDHA activity. In this test, LDH-A reduces NAD to NADH, which interacts with a specific probe to produce a color (λ_max_ = 450 nm), LDH-A level was measured using enzymatic colorimetric methods on Roche/Hitachi Cobas C systems (Integra 400 Plus, Germany). Results were expressed as percentage of LDH-A activity normalized to protein concentration.

### Western blotting

After treatment, cells were lysed and extracted the protein with protease inhibitor added RIPA lysis buffer. The protein concentration was measured by using BCA reagent. Proteins (50 μg) were resolved on polyacrylamide gels [29]. The gels were transferred into polyvinylidene difluoride (PVDF) membrane [30], and then blocked with 5% fat-free dry milk in PBS-T for 1 h. Blots will be then incubated with primary antibodies (anti- LDHA antibody-Rabbit monoclonal 1:1000, anti-pyruvate dehydrogenase - Rabbit monoclonal 1:1000, anti- VDAC antibody - Rabbit monoclonal 1:100, anti- Bax antibody - Rabbit monoclonal 1:1000, anti- cleaved caspase-3 antibody - Rabbit polyclonal 1:1000, anti- cleaved caspase-8 antibody - Rabbit monoclonal 1:1000, anti- cleaved caspase-9 antibody - Rabbit monoclonal 1:1000, anti- cytochrome C antibody - Rabbit monoclonal, 1:1000, anti-beta actin antibody Rabbit monoclonal, 1:1000, (Cell signaling technology, Danvers, MA, USA), and anti- Bcl2 antibody - Rabbit Polyclonal 1:1000, (Abcam, Cambridge, MA, USA) overnight at 4°C. Next day, blots were washed and then incubated with HRP-conjugated secondary antibodies and protein bands developed using ECL plus substrate (Thermo Pierce). Protein bands were visualized by a laser scanner (Typhoon FLA 9500, GE Healthcare Bio-Sciences AB, Sweden). Quantitation of protein was performed using the software Image J. Protein of interest was normalized against β actin to check equal loading.

### Immunofluorescent staining

Both HeLa cells and MCF7 cells were grown on coverslips in 6-well flat-bottom plates as non-treated wells and SO 60 mmol treated wells. Following the attachment of the cells to the coverslips and become confluent, treatment with SO 60 mmol will be started for 24 hours followed by washing the coverslips with PBS for 3 times then appropriate primary antibodies (anti-Bax antibody (Rabbit monoclonal, 1:50, Cell signaling technology, Danvers, MA, USA), anti- VDAC antibody (Rabbit monoclonal, 1:50, Cell signaling technology, Danvers, MA, USA), anti- cleaved caspase-3 antibody (Rabbit polyclonal, 1:50, Cell signaling technology, Danvers, MA, USA), anti- Bcl-xL antibody (Rabbit monoclonal, 1:50, Cell signaling technology, Danvers, MA, USA), anti-phospho-NF kappa B antibody (Rabbit monoclonal, 1:50, Abcam, Cambridge, MA, USA), anti-BMI-1 antibody (Rabbit monoclonal, 1:50, Cell Marque, Rocklin, CA, USA), anti- VEGF antibody (mouse monoclonal, 1:50, DAKO, Agilent, Santa Clara, CA, USA), anti- Ki-67 antibody (mouse monoclonal, 1:50, DAKO, Agilent, Santa Clara, CA, USA), will be added to the treated and untreated wells and kept for 24 hours. For double labeling a second primary antibody will be added for another 24 hour followed by washing. Then coverslips will be washed with PBS for 3 times and then Alexa Fluor 488 labeled donkey anti-rabbit antibody will be added for one hour followed by washing with PBS for 3 times. For double labeling Alexa Fluor 488 labeled donkey anti-rabbit antibody will be added for one hour followed by washing with PBS for 3 times and then Rhodamine labeled donkey anti-mouse antibody will be added followed by washing with PBS for 3 times. Afterward, coverslips will be mounted on slides using DAPI IF mounting media.

### Statistical analysis

The statistical analyses were performed using GraphPad Prism Software version 5. The comparisons between the various groups were achieved by one-way analysis of variance (ANOVA), followed by Newman-Keuls multiple range tests. Data were presented in mean ± standard error (S.E). *P* values < 0.05 were considered significant.
